# An Update on Drug–Nutrient Interactions and Dental Decay in Older Adults

**DOI:** 10.3390/nu15234900

**Published:** 2023-11-23

**Authors:** Victoria Bell, Ana Rita Rodrigues, Maria Antoniadou, Marios Peponis, Theodoros Varzakas, Tito Fernandes

**Affiliations:** 1 Faculty of Pharmacy, University of Coimbra, Pólo das Ciências da Saúde, Azinhaga de Santa Comba, 3000-548 Coimbra, Portugal; victoriabell@ff.uc.pt (V.B.);; 2 Department of Dentistry, School of Health Sciences, National and Kapodistrian University of Athens, GR-15772 Athens, Greece; mantonia@dent.uoa.gr (M.A.); mrpep@dent.uoa.gr (M.P.); 3 CSAP Executive Mastering Program in Systemic Management, University of Piraeus, GR-18534 Piraeus, Greece; 4 Food Science and Technology, University of the Peloponnese, GR-22100 Kalamata, Greece; 5 CIISA, Faculty of Veterinary Medicine, University of Lisbon, 1649-004 Lisbon, Portugal

**Keywords:** elderly, interaction, nutrients, medication, diet, oral health, decay, dental caries

## Abstract

In recent decades, the global demographic landscape has undergone a discernible shift that has been characterised by a progressive increase in the proportion of elderly individuals, indicative of an enduring global inclination toward extended lifespans. The aging process, accompanied by physiological changes and dietary patterns, contributes to detrimental deviations in micronutrient consumption. This vulnerable aging population faces heightened risks, including dental caries, due to structural and functional modifications resulting from insufficient nutritional sustenance. Factors such as physiological changes, inadequate nutrition, and the prevalence of multiple chronic pathologies leading to polypharmacy contribute to the challenge of maintaining an optimal nutritional status. This scenario increases the likelihood of drug interactions, both between medications and with nutrients and the microbiome, triggering complications such as dental decay and other pathologies. Since the drug industry is evolving and new types of food, supplements, and nutrients are being designed, there is a need for further research on the mechanisms by which drugs interfere with certain nutrients that affect homeostasis, exemplified by the prevalence of caries in the mouths of older adults. Infectious diseases, among them dental caries, exert serious impacts on the health and overall quality of life of the elderly demographic. This comprehensive review endeavours to elucidate the intricate interplay among drugs, nutrients, the microbiome, and the oral cavity environment, with the overarching objective of mitigating the potential hazards posed to both the general health and dental well-being of older adults. By scrutinising and optimising these multifaceted interactions, this examination aims to proactively minimise the susceptibility of the elderly population to a spectrum of health-related issues and the consequences associated with dental decay.

## 1. Introduction

The global increase in life expectancy presents multifaceted challenges, including nutritional considerations, diseases, and polypharmacy, all of which necessitate comprehensive examination to safeguard the health and well-being of the elderly [1]. Extensive research over several decades has explored the interactions between various foods/beverages and prescribed or non-prescribed drugs, uncovering side effects, alterations in drug bioavailability and nutrient absorption as well as their impact on conditions such as tooth decay [2]. While nutrients are fundamental units in nutrition, food/nutrient–drug interactions can arise from physical, biochemical, physiological, metabolic, or pathological interconnections between medications and food/nutrients [3]. Although these synergistic actions lack systematic study, understanding the potential interactions is pivotal for implementing dietary therapies or nutritional preventive approaches during pharmacological treatments [4,5]. The intricacies of this overview are heightened by imprecise nutrient requirement guidelines, ethical limitations on human trials, increased consumer expectations for quality and health outcomes, and the expanding market for food supplements and diet fortification promoted by influential food companies [6,7].

Global aging is a pervasive concern [8] and a paramount demographic shift in the 21st century [9]. Aging precipitates significant changes in physical, cognitive, and physiological realms, profoundly impacting nutritional needs. Aging brings declines in muscle mass, immune function, bone density, nutrient absorption, metabolism, and oral health status. This demographic encounters challenges in meeting the recommended nutrient requirements as food intake diminishes [10]. Additionally, reduced hunger stimulation, diminished sensations of thirst and taste, and potential hydro-electrolyte imbalances further hinder the attainment and maintenance of an ideal nutritional status [11,12].

An unbalanced diet, influenced by lifelong preferences, digestive changes, psychological factors, socioeconomic issues, disability, tooth loss, and prosthetic appliances, poses a risk of malnutrition in the elderly [13,14,15]. Dehydration, inherent to the aging process, contributes significantly to oral health problems, including xerostomia or dry mouth syndrome, which amplifies the likelihood of tooth decay [16]. Dry mouth, characterised by dysphagia, taste loss, and impaired appearance, not only impacts social lives but also leads to malnutrition, diminishing chewing ability, and causes nutrient deficiencies, limited food choices, poor diet quality, and compromised oral health [16,17]. Reduced salivary flow results in symptoms like burning, itching, chewing difficulties, taste impairment, and communication challenges [18,19], further correlating with issues such as difficulties wearing dentures, a poorer diet quality, and tooth decay [20,21]. Dental caries, stemming from oral bacteria’s fermentative actions on dietary sugars, introduces challenges in eating, pain, tooth loss, and a diminished quality of life [22]. Global surveys highlight widespread untreated caries, with peaks at 6, 25, and 70 years, the latter associated with increased root caries [23,24,25]. Key risk indicators for elderly caries encompass past root caries, surfaces at risk, poor oral hygiene, gender, age, periodontal disease, and nutritional discrepancies [15,26]. While fluoride has been effective, discussions increasingly stress the importance of diet and nutrient control for holistic oral disease prevention across all age groups, particularly among the elderly [27,28,29,30,31].

As aging progresses, numerous medications impact the nutritional status of the elderly, resulting in prevalent deficiencies in essential elements crucial for oral health and caries prevalence [32,33]. Precautions are vital to avoid interactions among medications, medications and foods (including beverages and dietary supplements), and medication, nutrients, and oral and gut microbiota [2]. The intricate five-way interaction—environment–host–drug–microbiota–nutrient—underscores the role of gut metabolites in energy metabolism, cell communication, host immunity, and various physiological activities, most notably aging [34]. These interactions, influenced by factors such as gender, age, body composition, nutritional status, clinical condition, and medication types and numbers, can manifest in various ways [35,36]. Chelation, a direct bio-transformation forming a non-absorbable complex between a drug and a food component, is one type of drug–nutrient interaction [37]. Such interactions pose the potential for either the augmentation or depletion of essential nutrients. Macroconstituents (proteins, lipids, and carbohydrates) provide molecular substrates for structural and metabolic processes, while micronutrients (vitamins and minerals) play crucial roles in maintaining optimal bodily function and the integrity of oral tissues. Given the aging body’s reduced ability to synthesise micronutrients, dietary supplementation becomes imperative in the presence of medications, requiring vigilant monitoring of nutrient intake levels [38,39,40,41].

This narrative review aims to address the theme of drug–nutrient interactions, particularly those associated with the commonly consumed medicines prevalent among the elderly population. Despite the vast array of drugs available on the market, research on the potential interactions influencing oral diseases like caries remains relatively limited.

## 2. Importance of Micronutrients in the Older Adults

The escalating life expectancy gives rise to a concomitant surge in the prevalence of chronic diseases, exerting inevitable ramifications on health, long-term care infrastructures, and overall quality of life [42]. Optimal well-being and nutritional status, it is asserted, should be cultivated well in advance of attaining senior age and prior to the irrevocable manifestation of diverse disease processes [43].

Micronutrients (e.g., omega-3 fatty acids, vitamins, and minerals—selenium, fluoride, zinc, iron, and manganese), are of particular importance and are fundamental in terms of human metabolism, physiology, and general and oral health. They participate in the maintenance of homeostasis and metabolism of the body, in the promotion and maintenance of health, and in the prevention of dental caries and other general diseases throughout our lifetime [44]. They participate in numerous biological processes, including the growth of bones, teeth, and tissues, synthesis of hormones and enzymes, cell and membrane repair, and reactions of energy metabolism, to mention a few [45]. Therefore, a deficit or excess of micronutrients can lead to the development of structural or functional changes and the occurrence of pathologies [46].

More specifically, the possibility of immunomodulation by certain foods has emerged, namely, the role of dietary glucans (β-1,3-glucans), from their bioactive role in various immune reactions and the treatment of cancer, and has been widely studied [47,48]. Consuming the recommended amount of β-glucans through diet may not cause side effects, but taking a β-glucan supplement may cause unintended adverse effects when interacting with medications that decrease the immune system’s response (immunosuppressants) [49]. Further, coenzyme 10 (CoQ10) is synthesised in the human body and is a substance naturally found (e.g., in the heart, liver, kidney, and pancreas) acting as an antioxidant protecting cells against oxidative stress and is involved in energy (ATP) production. Low levels of CoQ10 may be associated with older age, certain medications, genetic defects, nutritional deficiencies, and specific health conditions [50]. A deficiency of CoQ10 was found in inflamed human gingiva and has been found to be responsible for periodontal destruction and loss of teeth [51]. CoQ10, considered a nutraceutical, may make medications (e.g., statins and insulin) less effective at thinning the blood and interfere with antihypertensive, antiglycaemic, and chemotherapy drugs [52], resulting in oral inflammations that increase the risk of dental caries too [53].

Undoubtedly, the spectrum of metabolic interactions between drugs and nutrients is notably extensive. Within cellular structures, an array of highly specific signalling receptors exists, and drugs exert their influence on the cell membrane through physical and/or chemical interactions with these receptors, facilitated by chemical forces or bonds, often involving inner binding sites [54]. Notably, diets that induce acid formation contribute to urine acidification, thereby augmenting the elimination of alkaline drugs like amphetamines. Conversely, drugs with a base-forming nature enhance the elimination of acidic drugs, exemplified by barbiturates [55]. However, this domain is quite complex since it is impossible to study one binding interaction in isolation from the others at an interface. In practice, a synergistic response between non-covalent interactions and molecular responses is observed [56].

## 3. Older Adults and Polymedication

Population ageing is an increasingly evident and concerning demographic phenomenon. According to the World Health Organization, individuals aged sixty-five or older are classified as elderly in developed countries, while in developing countries, this age group is defined as those over sixty years old [57]. Population ageing is an increasingly evident and distinctive demographic phenomenon which is presently more important in developing countries [58]. The ageing process is associated with changes in physiology, metabolism, and body constitution, variations in nutritional status, and the development of various chronic pathologies along with polymedication [59]. The recent agendas of both climate change and healthy ageing are still open in an ageing global population. The impact of climate change on older adults’ health and well-being is an enormous public health concern and has been studied since uniquely vulnerable older people tend to have weaker immune systems, potentially posing a greater risk of severe infections and drug-induced nutritional deficiencies [60].

While a universally accepted definition remains elusive, the prevailing conceptualisation of polymedication commonly refers to the persistent, concurrent consumption of five or more medications. This encompasses a spectrum ranging from prescription drugs to non-prescription drugs, inclusive of those solely dispensed by pharmacies without prescription requirements, as well as dietary supplements [61,62]. Prolonged utilisation of medications and dietary supplements carries the potential to instigate both clinical and subclinical nutritional deficiencies. Awareness of drug–nutrient interactions is imperative not only for healthcare professionals but also for patients, as such knowledge safeguards against compromising the efficacy of medications and forestalls nutrient depletion. The latter, if unmitigated, may precipitate nutritional disorders, and malnutrition, and subsequently give rise to general and oral health complications. Therefore, active awareness and intervention directed toward mitigating the impact of such interactions are indispensable.

Certain drug-induced nutrient reductions are under discussion and subsequently escape diagnosis and treatment [21]. With the constant development of the pharmaceutical industry, new drugs appear for various therapeutic areas, proposing an improvement in the quality of life. However, the rational use of medicines is essential to optimise therapy and avoid nutritional deficiency. Therefore, it is essential to adapt a certain diet to each pharmacological therapy to achieve this rationale [63]. The elderly demographic emerges as the most vulnerable age group to the intricate dynamics of drug–nutrient interactions. This susceptibility stems not only from the prevalence of chronic and cumulative diseases, along with the concomitant conditions of undernutrition and impaired metabolism, but also from the heightened frequency of drug administration and the protracted duration of their treatment regimens [64].

## 4. Drug–Nutrient Interactions

A physical, chemical, and/or biological relationship is established between drugs and nutrients that can lead to the triggering of interactions. An interaction can be defined as the development of unexpected pharmacological or nutritional effects due to the joint administration of drugs and food, from which most of the nutrients originate. It should be noted that these interactions are not always clinically relevant, which does not mean that constant monitoring is not necessary [41].

Drug–food interactions can be broadly classified into two categories: direct physicochemical interaction and physiological or functional interaction. The drug–food interactions that elevate the plasma levels of drugs may originate advantageous or harmful therapeutic effects based on the potency and predictability of these interactions [42].

Considering the relationship between medicinal products and nutrients, interactions can be classified as (a) nutrient–drug interactions, in which the nutrient modifies or interferes with the pharmacokinetics and pharmacodynamics of the medicinal product, and (b) drug–nutrient interactions, in which the medicinal product affects the use and effect of nutrients by altering their bioavailability [43].

Drugs that stimulate intestinal peristalsis or modifiers of gastrointestinal secretions lead to the decreased absorption of nutrients [44]. It can also be a consequence of the adverse effects of drug. Certain medicines can cause changes in appetite, modify taste, and modulate the excretion of vitamins and minerals, thereby disturbing the level of micronutrients in the body [45]. Addressing the intricate phenomenon of drug–nutrient interaction, this review delves into the shifts in nutritional status resulting from specific drug administration. Recognised for its inter-individual variability in physiology and distinctions observed in special populations, this process is attributed to the modality of drug action, the influence on gut microbiota, and the resultant production of metabolites [65].

## 5. Host–Drug–Oral Microbiota–Nutrient Interactions

The human microbiota is the focus of one of the most dynamic current research fields, being widely acknowledged as a central regulator of host health; future technologies are anticipated to reveal the causation and molecular mechanisms yet undisclosed on this matter [66].

It is well known that the gut microbiome profile can be influenced by drugs, but, vice versa, the gut microbiota can also influence an individual’s response to a drug by enzymatically transforming the drug’s structure and altering its bioavailability, bioactivity, or toxicity [67]. The already complex issue is even more intricate when probiotics, prebiotics, synbiotics, paraprobiotics, postbiotics, and antibiotics are simultaneously administered [68]. The importance of a balanced gut microbiota for supporting optimal gut function and brain health and the possible impact of drug administration on the microbiota composition have also been highlighted. Drug–microbe interactions are still understudied in the clinical context, where, in the elderly, multiple drug use and comorbidities co-occur and how nutrient or food affects the gut ecosystem is still virtually undetermined [69].

The oral microbiota plays a major role in oral diseases such as periodontal disease or caries [70]. Both modalities are initiated by bacteria that accumulate in a biofilm on the tooth surface and affect the adjacent periodontal tissues. Oral microorganisms form a complex ecological community that influences oral and systemic health. Chronic diseases such as diabetes, rheumatoid arthritis, and systemic lupus erythematosus increase susceptibility to destructive periodontal disease and affect the prevalence of dental caries by enhancing inflammation. All three systemic diseases are linked to a decrease in bacterial taxa associated with health in the oral cavity and an increase in taxa associated with disease [71]. On the other hand, there is increasing supporting evidence that many chronic illnesses are associated with disturbances in the oral ecosystem, such as diabetes, cardiovascular diseases, and tumours [72]. Drugs prescribed for these systemic diseases also affect the oral microbiota and nutrient intake [31,73]. The impact of probiotics as well as of bacteria colonising food does not reside in their ability to graft in the microbiota but rather in sharing genes and metabolites, supporting the challenged microbiome, and directly influencing epithelial and immune cells all over the body and in the oral cavity. Such observations argue that probiotics could be associated with conventional drugs for insulin resistance, infectious diseases, inflammatory diseases, and psychiatric disorders and could also interfere with drug metabolism [74]. Up to now, it remains difficult to report with accuracy whether one would benefit from taking a particular probiotic together with drugs prescribed for the previously mentioned diseases and control dental caries and other oral diseases. There is a significant need for further research on the correlation of drugs for systemic diseases, the change in gut and oral microbiota due to changes in nutrient intake, and the use of natural sources of microorganisms that enhance the oral microbiota equilibrium such as probiotics, prebiotics, and symbiotics.

To further discuss this point, it is known that microbiota thrive on almost all dietary substrates. So, the nutrient fibres fermented by microbiota enzymes represent valuable prebiotics that specifically promote the growth of one or more bacterial types in the gastrointestinal tract and thus provide health benefits to the host in general, not only in the oral cavity. An excellent example is non-digestible carbohydrates from mushrooms like chitin and β-glucans. However, as mushroom supplements are commonly used as adjuvants to chemotherapy, there is an urgent need for clinicians and scientists to address the potential mushroom–drug interactions in oncology practice [75].

**Figure 1:**
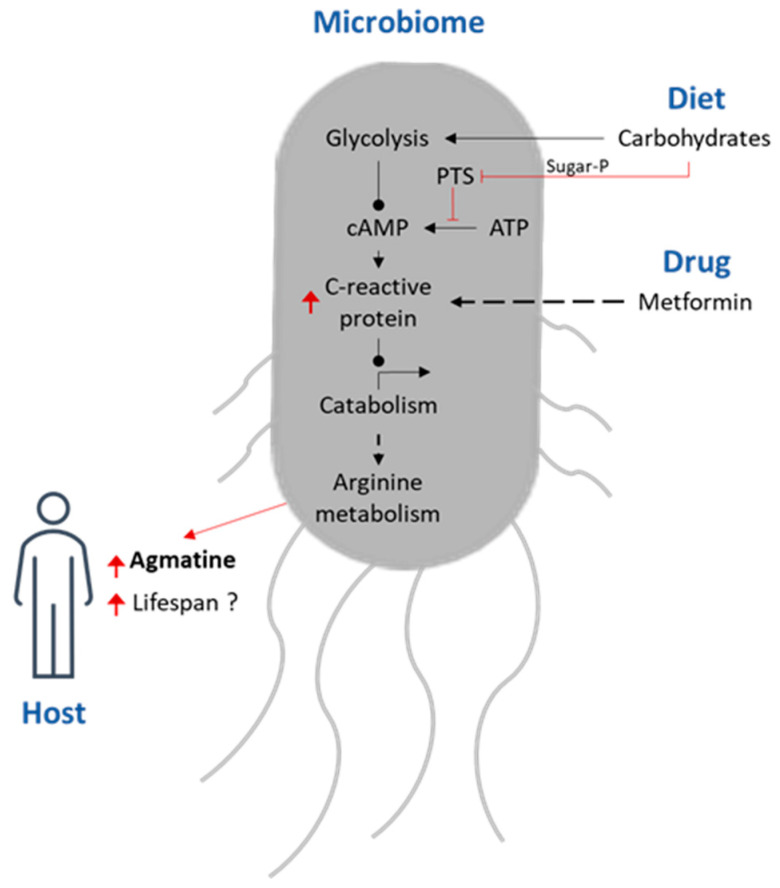
A model of the host–microbe–drug–nutrient interactions that regulate metformin’s effects on the host metabolism and lifespan.

In addition, agmatine is a member of biogenic amines and is an important medicine that is widely used to regulate body balance and neuroprotective effects [76]. A four-way host–microbe–drug–nutrient screening has been performed with a high-throughput method investigating host–microbe–drug–nutrient interactions with metformin, a very common drug used in type-2 diabetes. It was found that metformin host effects are linked and regulated by the bacterial nutrient agmatine signalling pathway (Figure 1) [77].

Another example is adenylyl cyclase which catalyses the conversion of adenosine triphosphate (ATP) to 3′,5′-cyclic AMP (cAMP) and pyrophosphate [78]. The phosphoenolpyruvate (PEP): carbohydrate phosphotransferase system (PTS) regulation network is found only in bacteria, with its catalytic function in sugar transport and phosphorylation; therefore, the microbiota plays a central role in metabolism and pathogenesis [79]. Metabolic syndrome (MetS), reflecting a cluster of metabolic abnormalities, has become the most important issue in family medicine and primary care, and high-sensitivity C-reactive protein (hs-CRP) levels and metabolic syndrome (MetS) are known to be associated with an increased incidence of different inflammatory situations and chronic diseases [78]. Pumpkin seeds and all other nuts, fish, and meat, have high concentrations of arginine, the nutrient that cancer cells crave. Increased L-arginine levels can exert pleiotropic effects on T cell activation, differentiation, and function. The beneficial effect of L-arginine on T cell survival and anti-tumour functionality may be exploited therapeutically, for instance, to improve adoptive T cell therapies [79]. However, L-arginine supplements can interact with some medications, such as blood thinners, nitro-glycerine, diabetes, and blood pressure drugs.

Overwhelmingly, the mechanisms of interaction between pathogenic and non-pathogenic microbiota and drugs used for different systemic diseases should be further explored in conjunction with natural supplements including microorganisms. Differences in the methodologies used so far in testing different strains, nutrients, and food beverages for controlling microbiota and the existence of newly designed drugs for systemic diseases describe an emergency in setting revised research protocols for a more holistic and natural control of these diseases.

## 6. Drugs Used for Basic Elderly Pathologies vs. Frequency of Dental Caries

In the aging process, the body undergoes alterations in the utilisation and absorption of medications, affecting the rate at which drugs traverse the gastrointestinal tract and the overall body. A deceleration in the circulatory system can prolong the time it takes for drugs to reach vital organs, consequently heightening the likelihood of interactions between various drugs as well as between drugs and nutrients (Table 1).

### 6.1. Antidyslipidemics–Statins

Statin drugs are used in the treatment of hypercholesterolemia and the prevention of certain cardiovascular diseases associated with atherosclerosis, such as stroke and acute myocardial infarction. They work by inhibiting the enzyme 3-hydroxy-3-methylglutaryl-coenzyme A reductase (HMG-CoA reductase), an enzyme involved in the endogenous synthesis of cholesterol [80]. Currently, there are seven statins available in the European market. They can be categorised as hydrophilic, such as rosuvastatin and pravastatin, and lipophilic, such as lovastatin, simvastatin, fluvastatin, pitavastatin, and atorvastatin. The latter is considered the most widely used active substance [81]. In the elderly population, cardiovascular disease is the most significant cause of morbidity and mortality, which is why statins are in high demand [82]. However, their use should be approached with caution because they interact with micronutrients like coenzyme Q10 and fat-soluble vitamins (A, D, E, and K), leading to their depletion in the body [83]. This fact has considerable effects on the oral cavity [84]. More specifically, a lower intake of vitamin A has been associated with decreased oral epithelial development, impaired tooth formation, enamel hypoplasia, and periodontitis. Vitamin D deficiency during tooth development may further result in non-syndromic amelogenesis and dentinogenesis imperfecta, enamel and dentin hypoplasia, and dysplasia. In addition, vitamin C deficiency results in changes in the gingivae and bone, as well as xerostomia; while vitamin B deficiencies are associated with recurrent aphthous stomatitis, enamel hypo-mineralisation, cheilosis, cheilitis, halitosis, gingivitis, glossitis, atrophy of the lingual papillae, stomatitis, rashes around the nose, dysphagia, and pallor. The effects of vitamins E and K on oral health are not as clear as those of other vitamins. However, a lack of vitamin K has a systemic effect (increasing the risk of haemorrhage), which may affect individuals undergoing oral surgery or suffering an oral injury [85].

**Figure 2:**
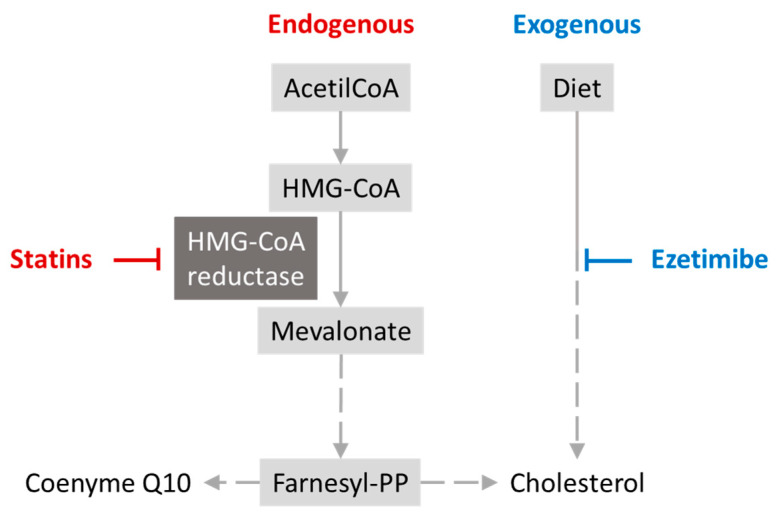
The action of statins in the mevalonate cycle.

As previously mentioned, coenzyme Q10 can be obtained endogenously through the mevalonate pathway. This pathway starts with Acetyl-CoA as the initial substrate, leading to HMG-CoA, then mevalonate, and other intermediates, ultimately producing cholesterol, dolichol, and coenzyme Q10 as end products [86]. Statins inhibit HMG-CoA reductase, the enzyme that catalyses the conversion of HMG-CoA to mevalonate, thereby reducing cholesterol production. However, by inhibiting this enzyme, they also decrease the production of coenzyme Q10. This mechanism explains the coenzyme Q10 depletion caused by statins, but the benefits outweigh the risks (Figure 2) [87].

On the other hand, statins are also involved in the reduction of fat-soluble vitamins, particularly a decrease in vitamin D. This class of drugs, by inhibiting HMG-CoA reductase, reduces the synthesis of various intermediates in the cholesterol metabolic pathway, such as 7-dehydrocholesterol (7-DHC), which is the endogenous precursor of vitamin D [88]. Therefore, reduced levels of this molecule can interfere with vitamin D levels. Thus, statin therapy, in addition to decreasing cholesterol production, can also contribute to the depletion of vitamin D [89,90]. There is also strong and plenty of evidence of the relationship between vitamin D deficiency and the development of myopathies [91]. It is known that statins are predominantly metabolised by the cytochrome P450 (CYP450) enzyme system, and vitamin D is an inducer of CYP3A4 and CYP2C9, aiding in the metabolism of certain statins. Since statins are associated with vitamin D depletion, a reduction in the metabolism of these drugs may occur, leading to an increase in their concentration and the consequent development of myopathies [92]. Further, clinical studies have demonstrated an association between vitamin D’s endocrine effects and periodontitis in the oral cavity. Overall, a significant association has been found between cariogenic activity and vitamin D deficiency in children [93,94] but also older people [95]. It has also been reported that vitamin D exerts anti-inflammatory effects and helps in calcium absorption and bone remodelling. Moreover, an adequate vitamin D status could reduce the formation of dental caries by delaying their onset and progression in the hard oral tissues as mentioned elsewhere [96].

Considering the above, it is essential to monitor the elderly during the chronic use of statins to prevent the deficiency of micronutrients which are crucial for general and oral health preservation.

### 6.2. Gastric Secretion Modifiers–Proton Pump Inhibitors

Antacids are bases that neutralise the acid in the gastric contents and are used to treat heartburn, gastroesophageal reflux, and peptic ulcers. Among these, are the proton pump inhibitors (PPIs) commonly used in the elderly population, known as “stomach protectors”, although they are really medications that inhibit the H+ and K+ ATPase of the stomach’s parietal cells [97].

PPIs contain active substances such as omeprazole, lansoprazole, pantoprazole, rabeprazole, esomeprazole, and dexlansoprazole. Within this therapeutic group, omeprazole and pantoprazole are the most commonly used active substances in Europe and the United States of America [98]. These drugs are often used for longer periods than recommended. Recent clinical studies indicate that prolonged use of PPIs can interfere with nutrient absorption, leading to some nutritional deficiencies, such as vitamin B12, magnesium, calcium, vitamin C, and iron, among others, with clinical implications [99].

Age is a potential risk factor for the development of vitamin B12 deficiency. Vitamin B12 (also known as cobalamin) is a B vitamin that has an important role in cellular metabolism, especially in DNA synthesis, methylation, and mitochondrial metabolism. Clinical B12 deficiency with classic haematological and neurological manifestations is relatively uncommon. Deficiency is caused by either inadequate intake, inadequate bioavailability, or malabsorption. The disruption of B12 transport in the blood, or impaired cellular uptake or metabolism, causes an intracellular deficiency causing recurrent aphthous ulceration, gum inflammations, and stomatitis [100,101,102].

The absorption of vitamin B12 involves the contribution of pepsin, which cleaves peptide bonds, separating vitamin B12 from dietary proteins [103]. Gastrin is a peptide hormone primarily responsible for enhancing gastric mucosal growth, gastric motility, and the secretion of hydrochloric acid (HCl) into the stomach. Initially, the enzyme pepsin is released in an inactive form, pepsinogen. When it meets HCL, it transforms into the active form, pepsin. After the separation of vitamin B12, it binds to intrinsic factor (IF), produced by gastric parietal cells, which prevents the pancreatic digestion of vitamin B12 and allows its absorption in the terminal ileum [104].

**Figure 3:**
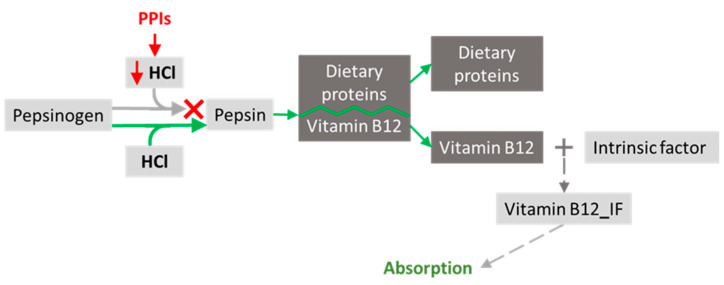
Long-term usage of proton pump inhibitors (PPIs) (e.g., omeprazole) is linked to an increased risk of vitamin B12 insufficiency in older patients, negative effects on the absorption of other vitamins and minerals, and decreased protein digestion.

Since PPIs are acid suppressors, there may be a reduction in the conversion of pepsinogen to pepsin, and, consequently, there will not be the separation of vitamin B12, which could lead to decreased absorption. Moreover, the elderly population is prone to IF deficiency [105]. These factors contribute to vitamin B12 deficiency in older adults taking PPIs. Indeed, there is a significantly increased risk of vitamin B12 deficiency in chronic PPI users (for more than continuous 12 months) compared to non-users or short-term users (Figure 3).

Prolonged use of PPIs has also been associated with hypomagnesemia [106]. This magnesium depletion may be associated with a decrease in active transport and changes in intestinal absorption, although its mechanism has not been fully clarified yet [107]. According to some observational studies, the administration of these drugs for a period of more than a year is significantly associated with a higher risk of developing hypomagnesemia [108]. In addition to depleting vitamin B12 and magnesium, PPIs also interfere with calcium levels. Studies have indicated that taking these drugs for 6–12 months is associated with an increased risk of hip, wrist, or vertebral fractures in the elderly [109]. The mechanism underlying these effects is not completely understood; however, it is possible that the decrease in hydrochloric acid (hypochlorhydria) induced by PPIs may lead to a reduction in calcium and vitamin D absorption [110]. Another hypothesis is related to the fact that these drugs can cause hypomagnesemia. This deficiency can induce dysfunction of the parathyroid glands, affecting the regulation of calcium levels [111]. Further, as known already from the relevant literature, minerals such as magnesium, calcium, and phosphorus found in the diet constitute the main structural components of the human tooth. Their inadequacy leads to absorption impairment, increased bleeding tendency, bone resorption, looseness, and premature tooth loss. Inadequacy of these essential minerals is associated with delayed tooth eruption and with enamel or dentin hypoplasia which creates a prosperous substrate for the development of dental caries. Taking calcium without magnesium results in soft dental enamel which cannot resist the acids that cause tooth decay [96].

Vitamin C is also a micronutrient that undergoes changes in the body with the use of PPIs. This vitamin, mainly in its reduced form (ascorbic acid), exists in high concentrations in gastric juice [151]. Ascorbic acid, when it reacts with free radicals or reactive oxygen species, is converted into its inactive form, dehydroascorbic acid (DHAA), and this reaction is reversible. However, DHAA can be hydrolysed into 2,3-diketogulonic acid at pH > 4 through an irreversible reaction [152]. Users who take PPIs reveal increased pH gastric levels and show reduced levels of ascorbic acid and total vitamin C in their gastric juice. The mechanism associated with the depletion of this antioxidant is not fully understood. However, it is likely due to the fact that vitamin C is unstable at a non-acidic pH and undergoes irreversible denaturation because DHAA can be hydrolysed to 2,3-diketogulonic acid, and the body cannot regenerate vitamin C from this compound [153]. The vitamin C pool in the body is usually depleted in 4 to 12 weeks if the intake of the vitamin is stopped. Ascorbic acid is affected by many factors that can impair absorption and its functions. The best way to prevent vitamin C deficiency in the elderly is to suggest them to consume fruits and vegetables regularly especially if they use PPIs [154].

Prolonged use of PPIs, by reducing gastric acid secretion and consequently increasing pH, also leads to decreased iron in the body. This iron exists in two forms: ferrous iron (heme iron) and ferric iron (non-heme iron), with ferrous iron being more easily absorbed. Gastric acidity promotes the conversion of ferric iron to ferrous iron [155]. Since PPI administration reduces gastric acidity, there is less conversion of ferric iron to ferrous iron, resulting in reduced iron absorption. This absorption, however, can be facilitated through the consumption, for example, of ascorbic acid (vitamin C) [112]. Another mechanism that may underlie iron deficiency due to the administration of PPIs is related to the hepcidin/ferroportin axis. Hepcidin is a small peptide synthesised in hepatocytes that acts as a controller of iron metabolism. It regulates the release of iron from enterocytes, hepatocytes, or macrophages (the main sites of iron absorption) into the blood. At the molecular level, hepcidin binds to the only cellular iron exporter, ferroportin, inducing its internalisation and subsequent lysosomal degradation. This leads to a decrease in the efflux of this mineral into the blood, resulting in iron deficiency anaemia [113]. Finally, another study demonstrated that PPIs increase the messenger RNA (mRNA) expression of hepcidin. The increased production of hepcidin by PPIs contributes to reduced ferroportin expression. Consequently, there is a reduction in iron absorption, leading to the development of iron deficiency anaemia. However, further studies are needed to confirm the effect of PPIs on hepcidin and its role in inducing anaemia [114].

Experiments in iron-deficient mice showed a higher risk of developing deep dental caries compared to non-anaemic mice, highlighting the protective role of iron against dental caries [115]. To further explore the issue in a recent systematic review and meta-analysis including clinical studies, it was revealed that iron deficiency was more prevalent in children with early childhood caries [116]. This should be further studied in the elderly population to come to specific recommendations when administrating PPIs. For all the above-mentioned reasons, very recently, the rational use of PPIs and general guidelines was formulated in India to promote the use of PPIs under appropriate indication and duration, helping clinicians to optimise their use in their practice [117].

### 6.3. Oral Hypoglycaemic Agent–Metformin

Metformin is a biguanide antidiabetic agent, with anti-hyperglycaemic effects, capable of reducing both basal and postprandial blood glucose levels [118]. It does not stimulate insulin secretion and therefore does not produce hypoglycaemia, being commonly used as a first-line option in monotherapy or combination therapy for the treatment of type 2 diabetes in the elderly [119]. This drug is based on three mechanisms of action: reducing hepatic glucose production by inhibiting gluconeogenesis and glycogenolysis, increasing insulin sensitivity in the muscles, thereby improving peripheral glucose uptake and utilisation, and delaying the intestinal absorption of glucose [120].

Observational and interventional studies show that prolonged use of metformin can negatively affect vitamin B12 and folic acid levels in the body. The mechanism by which metformin depletes vitamin B12 is not fully understood but is thought to be multifactorial. Primarily, several studies indicate that long-term therapy with metformin can affect the absorption of this vitamin through different mechanisms [121]. Metformin can reduce the secretion of intrinsic factor (IF) and can affect the calcium-dependent binding of the IF-vitamin B12 complex to the cubilin receptor (located in the microvillus membrane of enterocytes, in the distal ileum), a binding that is essential for the absorption of vitamin B12 [122]. It is also associated with a potential alteration in intestinal motility, which can lead to microbiota overgrowth, thereby blocking the absorption of the IF-vitamin B12 complex [122].

On the other hand, metformin can increase the amount of vitamin B12 normally stored in the liver, thus leading to a change in tissue distribution and metabolism of vitamin B12 [123]. Finally, it can also modify the metabolism and reabsorption of bile acids. Some of vitamin B12 is excreted in bile and goes through enterohepatic circulation to be reabsorbed [124]. By using metformin and causing alterations in bile acid reabsorption, less vitamin B12 may also be reabsorbed [125]. It should be noted that the deficiency in vitamin B12 induced by metformin is proportional to the duration of exposure and its cumulative dose [135] and has direct effects on oral caries status as mentioned before.

### 6.4. Central Nervous System (CNS) Medications–Antidepressants

Antidepressants are commonly used medications to treat depression and can also be used for other psychological conditions. There are various types of antidepressants, each acting differently and potentially causing different side effects [126]. A ten-year study conducted in Portugal to evaluate the evolution of anxiolytic and antidepressant consumption showed that, among antidepressants, drugs belonging to the class of selective serotonin reuptake inhibitors (SSRIs) were the most dispensed, with sertraline being the most consumed active substance [127]. Some studies have revealed an association between the use of SSRIs and the risk of developing osteoporosis. There is evidence that the consumption of these drugs can lead to the depletion of calcium and vitamin D, resulting in an increased risk of bone [128] and tooth fractures in the elderly [129].

SSRIs, as the name suggests, inhibit the reuptake of serotonin (5-hydroxytryptamine or 5-HT), a neurotransmitter that contributes to feelings of well-being and happiness and regulates psychological and behavioural functions such as mood, anxiety, and sleep. The inhibition of serotonin transporters (5-HTT) prevents the reabsorption of this neurotransmitter, leading to its accumulation in the synaptic cleft [130]. It is known that osteoblasts, osteoclasts, and osteocytes express 5-HTT [131]. When using an SSRI, it will also influence the 5-HTT in these cells. Therefore, some studies recommend calcium and vitamin D supplementation when using SSRIs [107] and when severe periodontitis coexists [136].

In a systematic review, the effects of SSRIs on bone mineral density (BMD) were found to be treatment time-dependent, causing a decrease in BMD with prolonged SSRI consumption at older ages [137]. Elsewhere it was suggested that SSRIs have a negative effect on bone formation via inhibiting osteoblast proliferation [132,133,134], although an in vitro study indicated that 5-HTT may be found not only in osteoblasts but also in osteoclasts [138]. As these cells have opposite effects on bones, the effect of SSRIs on bone metabolism seems to be more complex in vivo. Although the mechanism involved in this process is not yet completely understood, it seems that inhibiting 5-HTT in osteoblasts, osteoclasts, and osteocytes will have repercussions on both bone formation and resorption [139], resulting in periodontitis and the loss of teeth [136] or dental implants [140,141]. In a clinical study aimed to explore the relationship between SSRIs and the risk of failure in osseo-integrated dental implants, it was found that the failure rate was 4.6% in the nonuser group and 10.6% in the SSRI user group, meaning that SSRI users were more susceptible to implant failure as suggested elsewhere [142,143]. However, in none of these studies was a statistical significance level reached. Later, an extensive thesis on the effect of different types of antidepressants on dental implants showed that the SNRIs had the most significant implant failure rate, whereas SSRIs had the lowest failure rate among all the antidepressant types [144]. Interesting to mention is the fact that in addition to the antipsychotic effects of fluoxetines, a common antidepressant type, they were found to have anti-inflammatory, immunomodulatory, and additional analgesic effects resulting in inducing periodontitis and controlling other microbiota resulting in caries development [145,146].

Furthermore, the use of antidepressants, particularly SSRIs, is also associated with the risk of hyponatremia (low blood sodium levels). Risk factors for developing hyponatremia with SSRIs include advanced age, female gender, concomitant use of diuretics, low body weight, and lower baseline serum sodium concentration. Therefore, special attention should be paid to the elderly population, as they may be more susceptible to the risk of hyponatremia [147]. The process by which these drugs induce sodium depletion is not fully explained, but in many cases, hyponatremia appears to be a consequence of the syndrome of inappropriate antidiuretic hormone secretion (SIADH) [148]. It is thought that antidepressants may stimulate the release of antidiuretic hormone (ADH), possibly by increased serotonin activity, or they may increase the renal response to ADH. ADH, also known as vasopressin, primarily controls osmolality and body fluid volume. It acts on the kidneys to promote water reabsorption and increased sodium dilution [149]. Therefore, when using an SSRI, there may be an increase in water retention and, consequently, the development of hyponatremia [150].

### 6.5. Anti-Hypertensives

Hypertension is a public health problem and constitutes one of the most important risk factors for cardiovascular diseases. Its prevalence increases with age, with the elderly being the most affected age group. The adoption of a healthy lifestyle, such as regular physical exercise, weight control, salt restriction, a high intake of vegetables and fruits, and moderate alcohol consumption, may prevent or delay the onset of hypertension. However, most patients will also require pharmacological therapy, thus needing to resort to the administration of antihypertensive drugs for optimal control of this condition [156].

The association between antihypertensive drugs and the gut microbiome has been recently studied and a better awareness of the effects of pharmacomicrobiomics in the therapy of hypertension is contributing critical information for governing coherent clinical use and individualised use [157]. Angiotensin-converting enzyme inhibitors (ACE inhibitors), angiotensin receptor antagonists (ARAs), β-blockers, calcium channel blockers (CCBs), and diuretics are the five major classes of medications most commonly prescribed for this condition, recognised not only for their blood pressure-lowering effect but also for their risk-reducing effect [158].

The long-term use of anti-hypertension medications results further in xerostomia [159]. Dry mouth then raises caries prevalence in the mouth of the users. It is reported that elderly individuals who take these drugs for more than two years demonstrate a higher prevalence of root decay than those who do not [160].

It is known that food may affect the bioavailability of antihypertensive drugs, and this should also be carefully considered. The grapefruit juice–calcium channel blocker interaction is long-known and advising patients to remove grapefruit juice from their diet when receiving treatment with these drugs seems to be the best recommendation since the flavonoid and non-flavonoid components of grapefruit juice interfere with enterocyte CYP3A4 activity [161,162].

#### 6.5.1. Angiotensin Converting Enzyme Inhibitors (ACE)

ACE inhibitors, particularly medicament captopril used to treat high blood pressure, have interactions with important micronutrients, such as zinc, which can increase the risk of zinc deficiency [163]. This effect is more pronounced with the chronic use of captopril compared to other drugs in this class, especially when factors such as heart failure, kidney disease, advanced age, malabsorption, and diarrhoea are present [164].

The underlying mechanism may be associated with the presence of a thiol radical group in this drug, which can chelate serum zinc and, thus, increase its excretion. The depletion of zinc, an important element that supports many functions in humans, including the immune system, growth, and development, can lead to hypogeusia (reduced ability to taste), which can have serious consequences for nutritional status. Additionally, captopril treatment can induce a metallic taste, affecting appetite. Therefore, it is essential to be aware of this interaction and pay special attention to the development of such symptoms [165]. One limitation of the studies conducted is that plasma zinc levels do not always correlate with tissue levels. Therefore, studies encompassing a broader range of zinc levels in the body are needed to confirm whether these drugs indeed alter the distribution of zinc in tissues or its function [166].

Experiments in rats fed with a zinc-deficient diet showed that the zinc levels in bones and teeth were lower than those for rats fed supplemental zinc [167]. It is interesting to mention that increased dietary zinc seems to result in greater levels of zinc in bones and teeth, but the levels of calcium may decrease. Then, greater incidences of enamel carious lesions are observed. Therefore, dietary zinc may be an important trace mineral in the process of post-eruptive mineralisation of the enamel and may reduce the susceptibility of teeth to caries. Thus, zinc is a significant nutrient to caries development as proposed by clinical studies in humans too [168].

ACE inhibitors, especially captopril and enalapril, can also interfere with potassium levels, contributing to the development of hyperkalaemia (high blood potassium levels). These drugs inhibit the formation of angiotensin II, which stimulates aldosterone release in the adrenal glands. In turn, aldosterone stimulates sodium retention and potassium excretion. Thus, when using an ACE inhibitor, as it inhibits angiotensin II, there can be a decrease in aldosterone, and, consequently, a decrease in renal potassium excretion, leading to an increase in potassium levels in the body [169,170].

Potassium stands as a pivotal salivary buffer, contributing significantly to the buffering capacity of saliva. In the context of this salivary buffering capacity, the elderly may exhibit an increased susceptibility to the caries process, a correlation previously posited in the context of autistic children [171]. Notably, existing literature indicates that salivary electrolytes, including sodium, potassium, calcium, and phosphorous, assume a substantial role in influencing the prevalence of dental caries, particularly discernible in young diabetic individuals when juxtaposed with their non-diabetic counterparts, as well as in older adults with and without diabetes. Consequently, it appears that the impact of potassium deficiency on older patients may not be as pronounced [172].

#### 6.5.2. Calcium Channel Blockers (CCB)

Calcium channel blockers (CCBs) belong to a therapeutic class commonly used to treat heart and blood vessel conditions such as hypertension, angina, abnormal heart rhythms, and Raynaud’s phenomenon (a phenomenon which occurs due to the narrowing of the arteries in the hands and feet, causing painful and cold fingers) [173].

This class includes dihydropyridine derivatives (amlodipine and nifedipine) and non-dihydropyridine derivatives (diltiazem and verapamil). Dihydropyridines are commonly used to treat hypertension and effort angina and primarily block calcium channels in vascular smooth muscle, having a potent vasodilatory effect. Non-dihydropyridines have a weaker vasodilatory effect and preferentially block calcium channels in heart cells, thereby reducing heart rate. This helps control rapid heart rhythms such as atrial fibrillation [174].

CCBs may also interact with micronutrients in the body, including vitamin B9 or folic acid, which helps the body make healthy new red blood cells [175]. This interaction appears to result in the development of aphthous stomatitis [176] and gingival hyperplasia, with the highest number of cases reported after the use of nifedipine, with a dose-dependent risk. However, this condition has also been confirmed in patients using amlodipine [177].

Gingival hyperplasia manifests as an augmentation in the number of collagen fibres and is often linked to suboptimal oral hygiene practices. An additional plausible mechanistic explanation pertains to the diminished uptake of folic acid by gingival fibroblasts, a phenomenon likely attributed to the influence of nifedipine on this essential nutrient. Collagen synthesis is controlled by matrix metalloproteinases and tissue inhibitors of metalloproteinases. Degradation occurs extracellularly through the secretion of collagenases and intracellularly through phagocytosis by fibroblasts. In this context, CCBs, by reducing the absorption of folic acid by gingival fibroblasts, may lead to alterations in the metabolism of matrix metalloproteinases and the failure to activate collagenase. Consequently, there is an accumulation of collagen fibres and, thus, the development of gingival hyperplasia [178,179].

Additionally, folic acid deficiency is directly involved in initiating the cascade of events that trigger and intensify the cariogenic process [180] and is well-supported for early childhood caries [181]; this correlation has yet to be further explored for the elderly.

#### 6.5.3. Loop and Thiazide Diuretics

Loop and thiazide diuretics can also interfere with various micronutrients in the body [182]. Loop diuretics like furosemide inhibit the tubular reabsorption of electrolytes such as sodium, potassium, and chloride in both the proximal and distal tubules, as well as in the ascending limb of the loop of Henle. They do this by inhibiting the co-transporter system responsible for these ions (the Na-K-2Cl co-transporter) [183]. Thiazide diuretics like chlorthalidone, indapamide, and hydrochlorothiazide, on the other hand, work by inhibiting the NaCl co-transporter in the distal convoluted tubule [184]. Both types of diuretics, often used in the treatment of nephrolithiasis, result in increased excretion of sodium, chloride, potassium, magnesium, and zinc. They also affect calcium levels, although they have opposite effects on calcium regulation [185].

More specifically, chronic use of both loop and thiazide diuretics increases urinary potassium excretion, with thiazide diuretics causing more pronounced excretion. Loop diuretics directly inhibit potassium reabsorption in the loop of Henle, while thiazide diuretics stimulate renal potassium secretion through multiple mechanisms. Therefore, monitoring potassium levels is necessary when administering these drugs to prevent the development of hypokalaemia and associated issues [163,186].

Further, magnesium reabsorption primarily occurs in the ascending limb of the loop of Henle through passive paracellular transport and can also take place in the distal tubule through active transcellular transport. Loop diuretics may reduce the driving force of the Na-K-2Cl cotransporter, possibly decreasing paracellular magnesium transport. On the other hand, thiazide diuretics may reduce TRPM6 in the distal tubule, affecting magnesium transport. These mechanisms may explain increased magnesium excretion due to loop and thiazide diuretics [187].

Both types of diuretics mentioned in this paragraph can contribute to decreased serum zinc concentration, but special attention should be paid to thiazide diuretics. Some studies in humans have shown that the use of thiazides increases the urinary excretion of this mineral in individuals with hypertension, potentially leading to tissue zinc depletion [188].

Finally, the reabsorption of calcium can occur through passive paracellular transport in the proximal tubule and the ascending limb of the loop of Henle and through active transcellular transport in the distal tubule. Loop diuretics inhibit calcium reabsorption by reducing the driving force for paracellular calcium transport in the ascending limb of the loop of Henle, potentially leading to decreased calcium levels in the body. Low blood calcium levels (hypocalcaemia) trigger the parathyroid glands to produce parathyroid hormone (PTH) to increase calcium levels. Loop diuretics, by negatively affecting calcium homeostasis, can lead to secondary hyperparathyroidism [189].

The chronic use of loop diuretics has also been associated with an increased risk of fractures in the elderly due to increased urinary calcium excretion. While loop diuretics increase calcium excretion, the body may respond by increasing intestinal calcium absorption. However, in certain individuals, particularly the elderly, this compensatory mechanism may be less efficient, leading to decreased bone mineral density and an increased risk of fractures [190]. Regarding the oral cavity, patients on diuretic medication have a higher prevalence of xerostomia, periodontitis, dental caries, and mucosal lesions when compared with those who do not receive it [191].

In contrast to loop diuretics, chronic use of thiazide diuretics has been associated with the development of hypercalcemia due to their enhanced effect on renal calcium reabsorption. The population that appeared to be most susceptible to hypercalcemia was older Caucasian women. However, the frequency of hypercalcemia among thiazide diuretic users seems to be low [192]. While some observational studies indicate that thiazide diuretics may help protect against hip fractures, further research is needed before making definitive recommendations [193].

Several studies, both in animals and humans, highlight the potential of loop diuretics to increase urinary thiamine loss. These investigations, comparing thiamine excretion rates with urine flow rates, indicate that thiamine loss is associated with increased diuresis rather than the specific diuretic used [194]. Notably, studies on individuals with congestive heart failure (CHF) using a non-specific loop diuretic, furosemide, suggest a potential decrease in thiamine concentration due to urinary loss, possibly impacting cardiac performance in CHF patients [195]. Thiamine deficiency appears to escalate with higher furosemide doses, raising concerns for the elderly who are already at an increased risk of thiamine deficiency due to inadequate dietary intake [196].

#### 6.5.4. Potassium Sparing Diuretics (Triamterene) and Hydrochlorothiazide

According to older studies, the use of triamterene, a potassium-sparing diuretic, and hydrochlorothiazide, a thiazide diuretic, may be associated with decreased folate levels and the development of megaloblastic anaemia [197,198]. Triamterene, a medication used in the management and treatment of oedematous states, has a structure like folic acid, and in vitro studies have suggested that high doses of this diuretic could inhibit the enzyme dihydrofolate reductase. This enzyme is responsible for converting the inactive form of folic acid (dihydrofolate) into the active form (tetrahydrofolate), which is essential for nucleotide synthesis [199]. However, it would require high concentrations of triamterene for this enzyme inhibition to occur.

Folate, also known as folic acid, is a B vitamin that plays a crucial role in various metabolic processes, including DNA synthesis and repair, as already described. It is essential for the formation of red blood cells and overall cell growth [200]. Therefore, it is unlikely that healthy individuals are at risk of folate deficiency because they metabolise this drug rapidly. The main concern lies in the potentially toxic effects of the prolonged use of this diuretic in patients with alcoholic cirrhosis. In such cases, the metabolism of triamterene may be altered, leading to a greater inhibition of dihydrofolate reductase, a decrease in the active form of folic acid, and the development of megaloblastic anaemia [201]. Additionally, an observational study has established a relationship between the prolonged use of diuretics and reduced folate levels in the red blood cells of hypertensive patients. Moreover, a short trial conducted on hypertensive individuals showed a decrease in folate levels after six weeks of hydrochlorothiazide use [163].

These findings suggest that it is not only the administration of triamterene that negatively influences folate levels but also diuretics in general, especially thiazides, which may contribute to this decrease.

### 6.6. Corticosteroids

Corticosteroids (CSs) are synthetic analogues of cortisol and have anti-inflammatory and immunosuppressive effects. They are used to treat various inflammatory, allergic, and immunosuppressive conditions, and even some types of cancer [202].

Some of the notable CSs include prednisone, prednisolone, hydrocortisone, dexamethasone, methylprednisolone, and beclomethasone. However, this therapeutic class is associated with numerous adverse effects, including the development of osteoporosis [203]. The development and analysis of various studies have established a relationship between the doses of CSs administered, the loss of bone mineral density (BMD), and an increased risk of fractures in the hip and spine, regardless of age and gender. The data have shown an initial rapid decline in BMD, followed by a slower, progressive decline [204,205].

CSs impair the replication, differentiation, and function of osteoblasts and induce apoptosis in mature osteoblasts and osteocytes, leading to the suppression of bone formation. They also stimulate the formation of osteoclasts, contributing to increased bone resorption. This results in an imbalance between bone formation and destruction [206]. Additionally, these drugs reduce the intestinal absorption of calcium and increase urinary excretion. Therefore, these two mechanisms may explain the development of osteoporosis after prolonged corticosteroid administration [207]. Corticosteroid doses exceeding 5 mg per day and treatment periods longer than three months increase the risk of osteoporosis and fragility fractures. This negative effect is particularly pronounced in individuals who cannot consume the necessary amount of calcium and vitamin D in their diet and who are at an increased risk of bone fractures and osteoporosis, such as the elderly population [208].

The impact of the long-term use of CSs on the oral cavity has been reported, including an increased risk of periodontal disease and dental caries in addition to changes in the properties and function of saliva [209]. However, the impacts of CS use on oral health are unclear and may include a decrease in salivary flow, lower pH, and higher bacterial levels of *Streptococcus mutans* and *Lactobacilli* as well as an increased risk of dental caries [210]. These effects have been categorised based on CS administration routes, including topical formulation, with variable intensities. For instance, the median decayed, missing, and filled teeth (DMFT) score was found to be twofold higher in asthmatic patients on CS inhalers compared to non-CS users [211]. In addition, the levels of oral cariogenic bacteria were higher in asthmatic patients on CSs as reported in the literature. Combined with an increase in saliva acidity and decreased flow, these changes may significantly increase the risk for dental caries [4,5,6]. However, no consensus exists today on the best approach to manage dental patients currently on CS therapy [212]. Further, most anti-asthmatic medications contain, except for steroids, fermentable carbohydrates [213]. Therefore, frequent oral inhalation of these sugar-containing drugs, coupled with decreased salivary production, may contribute to an increased risk of dental caries. Previous research has shown that the duration of medication has a significant influence on the risk of developing dental caries in asthmatic patients, indicating that patients using salbutamol inhalers are at a higher risk of developing carious lesions compared with non-asthmatic patients [214]. Similarly, the frequency of use and dosing levels influence the prevalence of carious lesions. Studies have shown that children who use this type of medication more than twice a day experience more dental caries, and the risk increases if it is administered before bedtime [215]. Therefore, it is essential to prescribe fluoride supplements to patients receiving corticosteroids, especially those taking β-2 adrenergic agonists. Patients should also be educated to perform mouth rinses after using inhalers. Chewing sugarless gum for at least one minute after drug administration will also help to neutralise salivary pH, stimulate salivary flow, and buffer oral acids, reducing the risk of caries formation [209,216].

In general, limited sodium intake and the monitoring of potassium levels are recommended for individuals undergoing long-term corticosteroid treatment. A potassium-rich diet may initially be sufficient to maintain normal levels, but if this is not the case, potassium supplements may be required [217].

## 7. Overview

The elderly constitute a part of the population particularly susceptible to alterations in their ability to meet micronutrient requirements, thereby compromising both their physical capacities and social integration. This vulnerability stems from a myriad of factors, with polypharmacy representing a significant contributor to this prevailing and widespread circumstance. Consequently, this age group exhibits an elevated predisposition to host–drug–nutrient–oral microbiota interactions, culminating in the onset of specific complications in both systemic and oral health domains, notably exemplified by dental caries. Confronted with fluctuations in micronutrient levels, the elderly frequently resort to the consumption of dietary supplements as a strategic intervention to mitigate the impact of these alterations, introducing an additional determinant in the multifaceted interplay of factors influencing their health. Dietary supplements, typically derived from a myriad phytonutrients or fungal sources, while mandated to adhere to certain legal stipulations, are not held to the same rigorous regulatory standards as pharmaceutical medications [218,219]. Consequently, ensuring the prudent consumption of these supplements necessitates guidance from healthcare professionals who can assess and advise on potential interactions, a particularly imperative consideration for elderly consumers given their distinct physiological dynamics and potential susceptibility to complications. 

A concise approach to simplify this overview towards drug classes and incidence of dental caries is presented in Table 2.

Oral health management, specifically dental caries for the elderly in the polypharmacy stage, often compounded by xerostomia, requires a tailored approach to address the unique challenges they face as described above. Dental and medical healthcare officers should collaborate to provide practical guidance during dental or medical examinations conjointly as a team. Firstly, emphasising the importance of regular dental check-ups is crucial and should be performed by both groups. Educating patients on maintaining optimal oral hygiene, especially in the presence of xerostomia, is essential at any stage of consultation. Recommending toothbrushes with soft–medium bristles and fluoride toothpaste aids in preventing tooth decay while not traumatising the soft oral tissues. For those with xerostomia, saliva substitutes or artificial saliva products can provide relief and help maintain oral moisture [220]. Additionally, the benefits of non-fluoride products, such as calcium-phosphate and synthetic hydroxyapatites, have been highlighted as preserving mineral dental tissues [221]. An informative table with active agents and suitable oral care products containing chlorhexidine and fluoride [222], including examples like saliva substitutes and moisturising gels, can serve as a practical reference for healthcare professionals [223] and will need regular revision (Table 3). RCT clinical studies confirm the non-inferior effects of these products on hard dental tissues, supporting their inclusion in tailored oral health regimens for the elderly in polypharmacy with xerostomia [224].

A synoptic protocol for the use of these basic oral hygiene products for the elderly with polypharmacy is seen in Table 4 [220,221,222,223,224,225].

In conclusion, the existing literature is limited in providing comprehensive risk/benefit assessments of dietary supplementation amid significant changes in the micronutrient levels induced by chronic medication use affecting gut or oral microbiota and the incidence of dental caries. Furthermore, scant attention has been given to the potential and likely more extensive effects of ions present in food supplements, herbs, and over-the-counter products. Addressing this gap requires a commitment to conducting more high-quality observational and intervention trials to deepen our understanding of the clinical significance, potential consequences, and strategies to minimise the risks associated with drug interactions, particularly in the context of controlling oral diseases such as dental caries.

## 8. Conclusions

The elderly population is highly susceptible to micronutrient deficiencies, impacting both their physical capabilities and social integration. Polypharmacy significantly contributes to this vulnerability, increasing the likelihood of host–drug–nutrient–oral microbiota interactions. The elevated predisposition to these interactions among the elderly leads to specific complications in systemic and oral health, particularly dental caries. Faced with micronutrient fluctuations, the elderly often turn to dietary supplements as a strategic intervention to mitigate the impact of these alterations. However, the consumption of dietary supplements requires careful consideration due to their distinct physiological dynamics and potential susceptibility to complications. Dietary supplements, while mandated to adhere to legal stipulations, lack the rigorous regulatory standards applied to pharmaceutical medications. The prudent consumption of supplements necessitates guidance from healthcare professionals and is particularly crucial for the elderly given their unique physiological characteristics. When analysing host–drug–nutrient–oral microbiota interactions, the strength and quality of evidence are critical. There is a pressing need for more high-quality observational and intervention trials to understand the clinical significance, consequences, and strategies to minimise the risks associated with these interactions, especially in the context of oral diseases like dental caries, as well as revision of the protocols of oral hygiene guidelines to ensure the relevant situations coincide with polypharmacy in the elderly. Healthcare professionals, particularly pharmacists, medical doctors, and dentists, play a crucial role in advancing the understanding of host–drug–nutrient–oral microbiota interactions. This increased understanding equips professionals to actively participate in interventions, offer well-informed guidance, and initiate patient education with a focus on proactive prevention rather than reactive restoration measures.

## Figures and Tables

**Table 1:** Common drug–nutrient interactions [62,63,64,65,66,67,68,69,70,71,72,73,74,75,76,77,78,79,80,81,82,83,84,85,86,87,88,89,90,91,92,93,94,95,96,97,98,99,100,101,102,103,104,105,106,107,108,109,110,111,112,113,114,115,116,117,118,119,120,121,122,123,124,125,126,127,128,129,130,131,132,133,134].

Common Prescription Drugs		Nutrient
	Atorvastatin	
	Fluvastatin	
	Lovastatin	Coenzyme Q10
Statins	Pitavastatin	Vitamin D
[62,63,64,65,66,67,68,69,70,71]	Pravastatin	
	Rosuvastatin	
	Simvastatin	
	Dexlansoprazole	Vitamin B12
	Esomeprazole	Vitamin C
	Lansoprazole	Calcium
PPIs *	Omeprazole	Iron
[72,73,74,75,76,77,78,79,80,81,82,83,84,85,86,87,88,89,90,91]	Pantoprazole	Magnesium
	Rabeprazole	
Oral Hypoglycaemic Agents [92,93,94,95,96,97,98,99,100]	Metformin	Vitamin B12
		Vitamin D
SSRIs ** [101,102,103,104,105,106,107,108,109,110,111]		Calcium
		Sodium
ACE Inhibitors ***	Captopril	Zinc
[112,113,114,115,116,117]	Captopril/enalapril	Potassium
CCB ****	Amlodipine	Vitamin B9
[118,119,120,121,122,123]	Nifedipine	
		Vitamin B1
		Calcium
Loop Diuretics	Furosemide	Magnesium
[124,125,126,127,128,129,130,131,132,133,134]		Potassium
		Zinc
		Vitamin B1
	Chlorthalidone	Calcium
Thiazide Diuretics	Hydrochlorothiazide	Magnesium
[124,125,126,127,128,129,135,136,137]	Indapamide	Potassium
		Zinc
Thiazide Diuretics	Hydrochlorothiazide	Vitamin B9
[138,139,140,141,142]		
Potassium Sparing Diuretics	Triamterene	Vitamin B9
[138,139,140,141,142]		
Corticosteroids		Vitamin D
[143,144,145,146,147,148,149,150]		Calcium

* PPIs: proton pump inhibitors are commonly used to reduce the production of stomach acid. ** SSRIs: selective serotonin reuptake inhibitors are commonly used to treat depression and anxiety disorders. *** ACE inhibitors: angiotensin-converting enzyme inhibitors are commonly used to treat conditions such as high blood pressure (hypertension), heart failure, and certain kidney conditions. **** CCB: calcium channel blockers are used to treat various cardiovascular conditions.

**Table 2:** Association between drug classes and incidence of dental caries.

Drug Class	Frequency of Dental Caries
Antidepressants	High
Antipsychotics	Moderate
Antihypertensives	Low
Analgesics/opioids	High
Antihistamines	Moderate
Antacids	Low
Immunosuppressants	Moderate
Anti-diabetic Medications	Moderate
Antibiotics	Low
Corticosteroids	High

**Table 3:** Active agents and oral care products suitable for older patients.

Active Agents	Oral Care Products
Calcium-phosphate	Non-fluoride toothpaste and synthetic hydroxyapatite products
Fluoride	Fluoride toothpaste
Saliva Substitutes	Artificial saliva products
Sugar-free Chewing Gum	Xylitol-containing gum or lozenges
Alcohol-free Mouthwashes	Mild, alcohol-free mouthwashes
Antimicrobial Mouthwashes	Prescription-based mouthwashes with antimicrobial agents

**Table 4:** Oral hygiene protocol for the elderly with polypharmacy.

Protocol for the Long-Term Use of Chlorhexidine in the Elderly
Concentration and Frequency: - Use a lower concentration of chlorhexidine (e.g., 0.12%) to minimise side effects. - Limit the use of chlorhexidine to a short period, such as two weeks, with periodic breaks to prevent staining of the teeth and other side effects. - If prolonged use is necessary, consider using chlorhexidine on a maintenance schedule, such as a few times per week.
Oral Health Assessment: - Regularly assess the patient’s oral health to determine the continued need for chlorhexidine. - Monitor for any signs of adverse effects, such as tooth staining or changes in taste sensation.
Patient Education: - Educate the patient on the proper use of chlorhexidine, emphasising adherence to recommended concentrations and application frequency. - Highlight the importance of reporting any unusual side effects promptly.
**Protocol for the Long-Term Use of Fluoride in the Elderly**
Fluoride Toothpaste: - Encourage the use of fluoride-containing toothpaste with at least 1000 ppm (parts per million) of fluoride. - Ensure that patients are aware of the correct toothbrushing technique and the amount of toothpaste to be used.
Fluoride Mouthwash: - Recommend a fluoride mouthwash with a lower fluoride concentration for daily use. - In cases where a higher concentration is necessary, use should be intermittent to prevent potential side effects.
Professional Application: - Consider professional fluoride applications during dental visits, especially for individuals with a higher risk of dental caries. - Assess the patient’s overall fluoride exposure, including drinking water fluoridation, to avoid excessive fluoride intake.
Regular Monitoring: - Monitor the patient’s oral health regularly, including assessing the fluoride levels in drinking water, to adjust fluoride recommendations accordingly.
Patient Compliance: - Emphasise the importance of compliance with the prescribed fluoride regimen. - Address any concerns or issues related to the taste or discomfort associated with fluoride use.
**Protocol for Xerostomia Management in the Elderly**
- Stay hydrated by drinking water regularly. - Use saliva substitutes or artificial saliva products to alleviate dry mouth symptoms. - Use sugar-free chewing gum or lozenges that can stimulate saliva production. - Avoid caffeinated and alcoholic beverages, as they can contribute to dry mouth. - Choose mild, alcohol-free mouthwashes to prevent irritation. - Consider antimicrobial mouthwashes if prescribed by a healthcare professional. - Schedule routine dental check-ups to monitor oral health and address any emerging issues promptly.
